# Hematological Changes and Reference Intervals in Hanwoo Calves during the First 28 Weeks of Life

**DOI:** 10.3390/ani11061806

**Published:** 2021-06-17

**Authors:** Ui-Hyung Kim, Seung-Hwan Lee, Sang-Rae Cho, Sung-Sik Kang, Shil Jin, Jun-Sang Ahn, Soo-Hyun Lee

**Affiliations:** 1 Hanwoo Research Institute, National Institute of Animal Science, Rural Development Administration, Pyeongchang 25340, Korea; sskang84@korea.kr (S.-R.C.); chosr@korea.kr (S.-S.K.); jins21@korea.kr (S.J.); dkswns121@korea.kr (J.-S.A.); 2 Division of Animal & Daily Science, Chungnam National University, Daejeon 34148, Korea; uhkim@korea.kr; 3 Animal Breeding and Genetics Division, National Institute of Animal Science, Rural Development Administration, 114, Sinbang 1-gil, Seonghwan-eup, Seobuk-gu, Cheonan 31000, Korea; slee45@cnu.kr

**Keywords:** calf, CBC, sex, Hanwoo, Hemavet 950

## Abstract

**Simple Summary:**

Complete blood cell count is important in identifying diseases in animals, and reference intervals must be established considering physiologic differences in genotype, age, sex, and management. However, little research has been performed to evaluate complete blood cell count reference intervals in Hanwoo calves. Therefore, we compared the complete blood cell count reference intervals of Hanwoo cows and Hanwoo calves and examined changes in calves’ complete blood cell count from birth to 28 weeks. We confirmed that complete blood cell count reference intervals specifically for calves are necessary for accurate diagnosis of calf diseases. In addition, the difference in complete blood cell count between female calves and male calves was confirmed for only some ages and some parameters. Our results suggest that reference intervals for Hanwoo calf complete blood cell count are necessary for accurate diagnosis of calf diseases

**Abstract:**

Hematological reference intervals must consider several parameters, including genotype, age, sex, management, and analytic process. Work is needed to evaluate hematological changes specifically in Hanwoo calves and according to calf sex. Therefore, in this study, we sought to confirm the complete blood cell count (CBC) reference intervals in Hanwoo calves, to monitor changes in hematologic values in Hanwoo calves from birth until 28 weeks of life, and to compare the hematologic values of male and female calves. A total of 35 male calves and 35 female calves was studied. Calf blood was sampled at multiple intervals from the time of birth until 28 weeks of age (including within 6 h of birth and at 2 days, 7 days, and 4 weeks and then at 4-week intervals through 28 weeks). In addition, blood samples were collected from 210 clinically healthy pregnant Hanwoo cows to establish CBC reference intervals for adult cattle. There were significant differences in the results of the cows and calves in all 14 parameters considered. The CBC reference intervals of the calves were wider than those of the cows in all parameters except mean corpuscular volume, mean corpuscular hemoglobin concentration, and mean platelet volume. We also identified differences from birth through 28 weeks between male and female calves at only some ages and some parameters. These results suggest that CBC reference intervals specific to Hanwoo calves are necessary for accurate diagnosis of calf diseases.

## 1. Introduction

Complete blood cell count (CBC) evaluation is important in identifying diseases in animals [1,2,3,4,5]. Laboratory diagnoses often are made by comparing an animal’s values to reference intervals from clinically healthy animals [5,6]. This method of diagnosis is dependent upon the use of correct reference intervals. Therefore, CBC reference intervals must be established considering physiologic differences in genotype, age, sex, and management [5,7]. Brun-Hansen et al. [8] found that common CBC reference intervals for cattle are based upon samples from adult animals and suggested that these reference intervals can be misleading when used to evaluate young calves.

Hanwoo is an indigenous Korean beef cattle breed, derived from *Bos taurus* [9]. Establishment of accurate CBC reference intervals for Hanwoo cattle is necessary for animal welfare and research. The most dangerous diseases in Hanwoo cattle include gastrointestinal or respiratory diseases that occur during the calf period [10,11]. Kim et al. [12] found that 87% of calf deaths were caused by gastrointestinal and respiratory diseases and occurred within 12 weeks of birth. To diagnose and treat calves, accurate, age-appropriate CBC reference intervals are essential. In addition, CBC is used as a parameter for assessment of physiological status in nutrition and metabolism studies of cattle [13,14,15,16]. However, little research has been performed to evaluate CBC reference intervals in Hanwoo calves and adults.

Therefore, in this study, we confirmed existing CBC reference intervals for adult Hanwoo cows and compared these values to those for Hanwoo calves. In addition, hematologic values were monitored continuously in 70 calves (35 males and 35 females) from within 6 h of birth to 28 weeks of age and compared between male and female calves.

## 2. Materials and Methods

### 2.1. Animals

A total of 80 Hanwoo calves was used in this study. All calves were born at a farm in Daegwllyeong, Gangwondo, South Korea. The sample included 40 males and 40 female calves. Animal health and welfare practices followed approved guidelines of the Animal Care and Use Committee (NIAS). The Ethics Committee approval number for this study was NIAS2021-504. All pregnant cows were housed in separate delivery stalls for one month before delivery. Starting at 1 week after birth, the calves were allowed access to concentrate, and hay was provided ad libitum until the age of three months. The calves were weaned at three months of age, and male and female calves were separated. Groups of 10 calves were transferred into pens and fed a standard diet of concentrate (crude protein 17%, crude fiber 8%, ether extract 3%, and crude ash 7%), hay, and a mineral supplement. The calves were dehorned before the age of 3 weeks. All male calves were castrated at the age of 6 months. The minimum time from dehorning to sampling was 3 days and from castration to sampling was 7 days. To establish reference intervals for adult cattle, blood samples were obtained from 210 clinically healthy Hanwoo cows (calving number 1–5) at the same farm. At all blood sampling times, a veterinarian was present and blood sampling was started after verifying the health of cows and calves.

### 2.2. Blood Sampling

Blood was collected from the calves at the following ages: within 6 h of birth, 2 days, 7 days, 4 weeks, 8 weeks, 12 weeks, 16 weeks, 20 weeks, 24 weeks, and 28 weeks. Any calf that demonstrated clinical signs of disease at the time of blood sampling was excluded. Ten calves (five male and five female) were excluded from final statistical analysis because of diarrhea or pneumonia at the time of sampling.

On each day of sampling, 3 mL of blood was collected from the jugular vein from each of the 80 Hanwoo calves into evacuated tubes containing K_3_EDTA (BD Vacutainer^®^, Franklin Lakes, NJ, USA). The 210 clinically healthy Hanwoo cows were all pregnant at the time of sampling. Three milliliters of blood were collected from the jugular veins of these cows into evacuated tubes containing K_3_EDTA.

### 2.3. Hematologic Analysis

The blood samples were stored at room temperature (approximately 20 °C) and were analyzed within 4 h of sampling. Hematologic parameters were measured using a Hemavet 950 (Drew Scientific, Waterbury, CT, USA): red blood cell count (RBC); hematocrit (HCT); hemoglobin (HGB); mean corpuscular volume (MCV); mean corpuscular hemoglobin concentration (MCHC); red cell distribution width (RDW); white blood cell count (WBC); absolute numbers of neutrophils (NE), lymphocytes (LY), monocytes (MO), eosinophils (EO), basophils (BA), and platelets (PLT); and mean platelet volume (MPV).

### 2.4. Data Analysis

The data for hematological parameters were tested for normality using the Shapiro–Wilk Test with *p*  >  0.05. Significant differences between young and old animals were investigated using Student’s t-test. Time-series hematologic parameters were plotted between males and females, and significant differences were tested by Student’s t-test at each time point. The reference interval was calculated based on a 95% confidence interval. Statistical analyses were performed using R software version 4.0.5 (R Core Team, Vienna, Austria) [17].

## 3. Results

**Figure 1:**
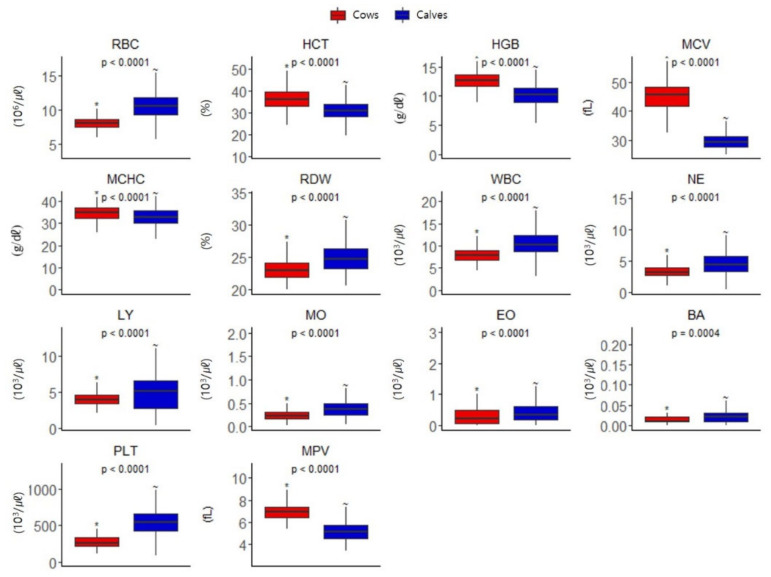
Complete blood cell count comparison of 210 pregnant Hanwoo cows and 70 Hanwoo calves (from within 6 h of birth to 28 weeks, a total of 700 samples).

The measured hematologic parameters of 210 pregnant Hanwoo cows and 70 Hanwoo calves (from within 6 h of birth to 28 weeks, a total of 700 samples) were compared. There were significant differences in the results of cows and calves for all 14 parameters (Figure 1).

The CBC reference intervals for Hanwoo cows and calves (from birth to 28 weeks) are shown in Table 1. For all parameters except MCV, MCHC, and MPV, the calves’ reference interval was wider than the cows’ reference interval. For three parameters, WBC, LY, and BA, the cows’ reference interval was included within the calves’ reference interval. The cows’ reference interval for parameters RBC, RDW, NE, MO, EO, BA, and PLT mostly overlapped with that of the calves. However, for MCV, only a small portion of the intervals overlapped.

**Figure 2:**
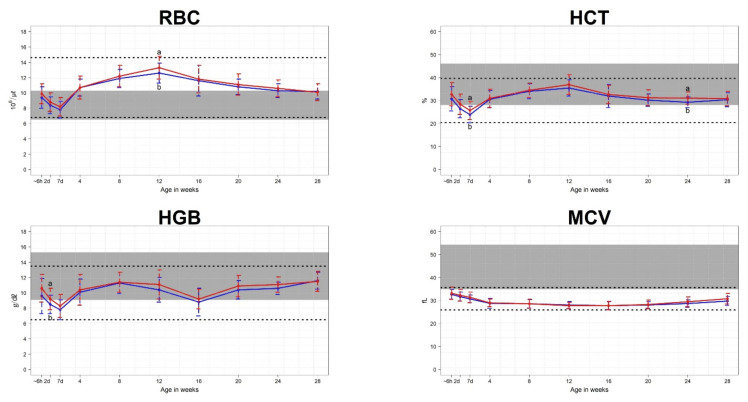

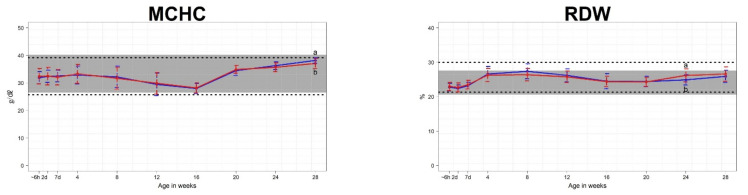
Means and standard deviations for erythrocyte parameters in 70 Hanwoo calves (35 male and 35 female). The blue line represents the mean for male calves, and the red line represents the mean for female calves. The shaded areas show the reference intervals for Hanwoo cows. The areas between the lower and upper dashed lines represent the reference intervals of Hanwoo calves. The x-axis displays the sampling ages, where ~6h is within 6 h of birth, 2d is 2 days after birth, 7d is 7 days after birth, and the numbers 4–28 correspond to the number of weeks after birth. a,b: Indication that mean values within the same age are significantly different (*p* < 0.05).

**Figure 3:**
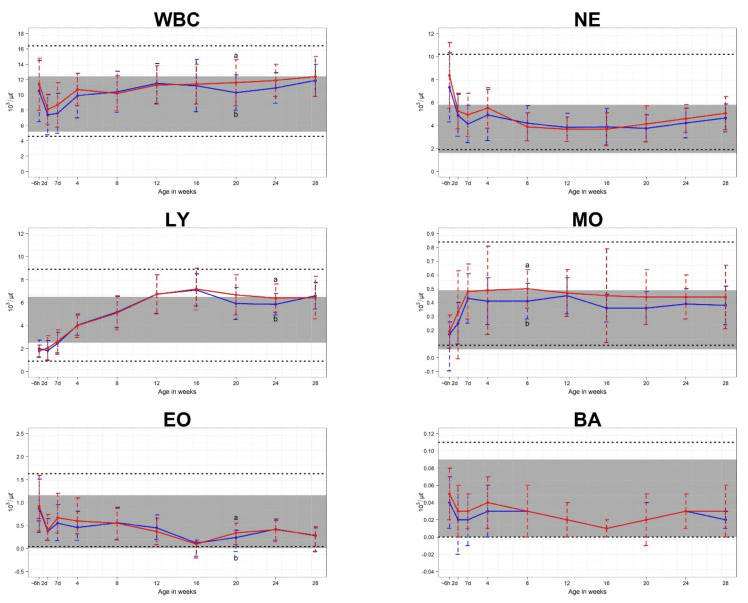
Means and standard deviations values for leukocyte parameters in 70 Hanwoo calves (35 male and 35 female). The blue line represents the mean for male calves, and the red line represents the mean for female calves. The shaded areas show the reference intervals for Hanwoo cows. The areas between the lower and upper dashed lines represent the reference intervals of Hanwoo calves. The x-axis displays the sampling ages, where ~6h is within 6 h of birth, 2d is 2 days after birth, 7d is 7 days after birth, and the numbers 4–28 correspond to the number of weeks after birth. a,b: Indication that mean values within the same age are significantly different (*p* < 0.05).

**Figure 4:**
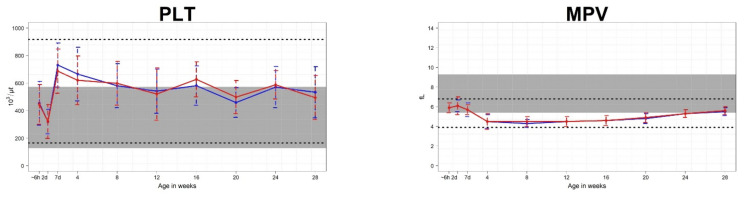
Means and standard deviations values for platelet parameters in 70 Hanwoo calves (35 male and 35 female). The blue line represents the mean for male calves, and the red line represents the mean for female calves. The shaded areas show the reference intervals for Hanwoo cows. The areas between the lower and upper dashed lines represent the reference intervals of Hanwoo calves. The x-axis displays the sampling ages, where ~6 h is within 6 h of birth, 2d is 2 days after birth, 7d is 7 days after birth, and the numbers 4–28 correspond to the number of weeks after birth.

Mean CBC parameters were compared for each age from birth to 28 weeks. For erythrocyte parameters, the following values differed for male and female calves: RBC at 12 weeks, HCT at 7 days and 24 weeks, HGB at 2 days, MCHC at 28 weeks, and RDW at 24 weeks (Figure 2). For leukocyte parameters, the following values differed for male and female calves: WBC at 20 weeks, LY at 24 weeks, MO at 8 weeks, and EO at 20 weeks (Figure 3). For platelet parameters, there were no differences between male and female calves (Figure 4).

## 4. Discussion

To produce highly marbled beef, male Hanwoo calves are castrated at about 6 months and raised until about 30 months. With Hanwoo steers, disease rarely occurs after the calf period; however, the risk of various diseases is higher with female Hanwoo because, due to calf production, females are raised for a longer period of time than male Hanwoo. Therefore, in this study, we confirmed existing CBC reference intervals for healthy pregnant Hanwoo cows and compared these values to reference intervals for Hanwoo calves.

In this study, when comparing the CBC results of the 210 Hanwoo cows and 70 calves, there were significant differences in all 14 parameters. In particular, MCV showed different patterns in cows (45.3 ± 4.7 fL) and calves (29.7 ± 2.6 fL). These results indicate that the CBC reference intervals of cows and calves have a different pattern, depending on the specific parameters.

Some of the CBC reference intervals that we characterized in Hanwoo cows were comparable to those identified in dairy cows, while others were not [1,4,5,8,18]. In particular, the reference intervals for RBC, HCT, and HGB in Hanwoo cows were higher than those of dairy cows [4,5,8,18]. Wood and Quiroz-Rocha [19] found that beef cattle have higher RBC values than dairy cattle breeds, similar to our results. For 11 parameters except MCV, MCHC, and MPV, the widths from the lower boundary to the upper boundary of the CBC reference intervals of calves were larger than those of the cows. Most of the reference interval regions of cow parameters were included or overlapped with calves’ reference intervals regions, except for HCT, HGB, MCV, and MPV. These results are due to CBC changes from birth to 28 weeks in calves. In particular, the changes in each parameter were most severe within the first 4 weeks after birth. Mohri et al. [3] reported that many values vary with age of the animal, with major changes occurring before puberty, and Knowles et al. [8] reported frequent large changes in variables associated with normal growth. These results suggest that the CBC results of a normal calf can be misdiagnosed as a disease if compared against the reference interval for adult cows. Therefore, calf CBC reference intervals are necessary for accurate diagnosis of diseases.

We found that mean RBC, HCT, and HGB tended to decrease until 7 days after birth, after which they increased until ~12 weeks. These results were similar to those of a previous study [8]. Tennant et al. [20] explained that rapid decreases in RBC, HGB, and HCT in neonatal calves are related directly to alterations in body fluid and the plasma volume expansion that follows colostrum ingestion. They also reported that subsequent decreases in these values were caused by the inability of the calf to produce erythrocytes at a rate equal to that of their removal from circulation. We found that the mean MCV decreased during the first 8 weeks of life and consistently remained below the cow reference interval through the 28th week. These results agree with results from a prior study [8]. Tennant et al. [20] reported that, although there can be differences in duration of MCV decline after birth, MCV values are uniformly lowest in young calves and progressively increase after 6 months of age to normalize by 24 to 30 months. A previous study [8] reported that the declining MCV is explained by replacement of RBCs containing fetal hemoglobin with smaller RBCs containing adult hemoglobin, and the lower MCV is compensated by higher RBC numbers to maintain normal HGB concentration. Mean MCHC and RDW values stayed within the cow reference intervals from birth to 28 weeks of age.

The mean WBC decreased for the first two days of life and then increased to the upper limit of the cow reference interval until 28 weeks of life. After birth, the mean neutrophil count was higher than the cow reference interval and decreased for the first 7 days of life. After this, the neutrophil count stayed within the cows’ reference interval to 28 weeks of life. The mean lymphocyte count was below the cows’ reference interval after birth and then increased for the subsequent 16 weeks of life. Mohri et al. [3] explained that an increased concentration of cortisol in neonates leads to higher numbers of WBCs and neutrophils and lower numbers of lymphocytes at birth. The mean monocyte, eosinophil, and basophil counts stayed within the reference intervals for cows from birth through 28 weeks of life.

The mean platelet counts decreased by the second day after birth, increased rapidly until the seventh day, and gradually decreased into the cows’ reference interval until 28 weeks. This pattern is similar to that found in a previous study [8]. The mean MPV decreased until 4 weeks after birth and then was maintained below the cows’ reference interval from 4 weeks to 28 weeks of life. The result that the MPV of calves is lower than that of cows has been reported in previous studies [21]

Panousis et al. [5] reported that neonatal female Holstein calves had a significantly higher erythron profile than males, while no differences were observed for leukocyte and platelet parameters. Raleigh and Wallace [22] reported that, in Hereford cattle, the HGB concentration was significantly higher in heifer calves than in steer calves from birth to 25 weeks of age. However, in the present study, the following erythrocyte parameters of female calves were higher than those of male calves: RBC at 12 weeks, HCT at 7 days and 24 weeks, HGB at 2 days, and RDW at 24 weeks. For MCHC, the values for male calves were higher than those for female calves at 28 weeks. For leukocyte parameters, the following values for female calves were higher than those of male calves: WBC at 20 weeks, LY at 24 weeks, MO at 8 weeks, and EO at 20 weeks. When looking at the overall changes in CBC count from birth to 28 weeks, these differences between female and male calves existed for only a portion of the calf period. Therefore, according to the results of this study, additional research is needed to determine whether the CBC reference interval required for accurate diagnosis of calf disease from birth to 28 weeks needs to be classified according to sex.

## 5. Conclusions

In this study, the results indicated that calf CBC reference intervals are necessary for accurate diagnosis of calf diseases. Unlike in previous studies, CBC differences between female calves and male calves were confirmed for only some ages and some parameters. However, the Hanwoo calves’ reference intervals of this study should be supplemented with the following additional studies to be used for accurate diagnosis of calf diseases. In this study, the CBC reference intervals were confirmed for the entire period from birth to 28 weeks, in 70 calves. However, calves should be divided according to ages when the parameters increase, remain constant, and decrease, providing the CBC reference interval for each age. Furthermore, studies are necessary to determine whether CBC reference intervals are required according to calf sex, either in the entire calf period or for only a portion.

## Figures and Tables

**Table 1:** Reference intervals for 210 pregnant Hanwoo cows and 70 calves (from within 6 h of birth to 28 weeks, a total of 700 samples).

Parameter	Unit	Cows	Calves
RBC	10^6^/µℓ	6.5–10.3	6.8–14.6
HCT	%	28.1–46.1	20.4–39.7
HGB	g/dℓ	9.1–15.3	6.5–13.5
MCV	fL	34.9–54.3	25.9–35.7
MCHC	g/dℓ	26.5–40.2	25.7–39.2
RDW	%	20.6–27.6	21.3–30.0
WBC	10^3^/µℓ	5.2–12.4	4.6–16.4
NE	10^3^/µℓ	1.6–5.8	1.9–10.2
LY	10^3^/µℓ	2.5–6.5	0.9–8.9
MO	10^3^/µℓ	0.06–0.49	0.09–0.84
EO	10^3^/µℓ	0.01–1.16	0.04–1.63
BA	10^3^/µℓ	0.00–ℓ0.09	0.00–0.11
PLT	10^3^/µℓ	129–573	166–918
MPV	fL	5.4–9.3	3.9–6.8

## Data Availability

The data presented in this study are available on request from the corresponding author.
